# Supplementary material for: Characteristics of ChatGPT users from Germany: Implications for the digital divide from web tracking data
Source: PLoS One. 2025 Jan 17;20(1):e0309047. doi: 10.1371/journal.pone.0309047 (PMC11741609; doi:10.1371/journal.pone.0309047)
Supplement: S1 File — (PDF) [file pone.0309047.s001.pdf]

Supplementary Materials for research paper entitled “Characteristics of users of ChatGPT from Germany” – Kacperski et al., 2024

Contents

S1. Survey details 2

S1.1. Survey items – full list before feature reduction 2

S1.2. Survey items – full correlation table 7

S2. Demographics distribution of sample (age, gender, education crossed). 8

S3 Web-tracking information 9

S3.1 Text Domain visits explained 9

S3.2 ChatGPT visits and adoption Distribution 10

S4. Non-imputed regressions 11

S5. Feature Selection 13

S5.1. Histograms of iteration in which features are selected by LASSO 13

S5.2. Examples of cross-validation curves in LASSO iterations 15

S5.3. Examples of coefficient plots in LASSO iterations 18

S6. Examples of Receiver Operating Characteristic (ROC) curves for ChatGPT Usage 21

S7. Data checks 23

S7.1 Correlations Tables 23

S7.2 Variable Inflation Factor 26

S8. Zero-Inflation Models 27

S1. Survey details

S1.1. Survey items – full list before feature reduction

All items here are translated from the original German version. For that version, please contact the authors. Variables with an option corresponding to 0 usage were coded 0, all variables with an option of no answer or don't know were coded as NA. Only in knowledge items, were 1 indicated correct answer, "don't know" was coded as 0. Some variables were recoded to simplify analysis (educational levels, employment), this is indicated in the column Analysis metrics.

S1 Table. Overview over survey constructs used in the lasso regression feature selection.

Overview	Analysis metrics
**Gender (gender)**	
Please state your gender 1 male 0 female 3 diverse (namely: [open answer field])	
**Age (age)**	
Please state your age open text field numeric 18-99	
**Education years (education)**	
How many years did you attend school in total, including any attendance at a vocational school or university? Please take into account all full and part-time training and convert the total duration of your schooling or training into whole years.<br>1 open answer field numerical information from 0-98	
**Education degree (degree, edu_s)**	
What is the highest general education school or university degree that you have achieved?<br>1 Elementary school not finished 2 Elementary school finished, but not (yet) a qualification from a secondary school 3 Elementary school/secondary school certificate or polytechnical high school with grade 8 or 9 4 Middle school certificate/Realschule certificate or polytechnic high school with grade 10 5 Technical college entrance qualification (Graduation from a technical college, etc.) 6 Abitur or advanced high school with a 12th grade (university entrance qualification) 7 Intermediate examination, pre-diploma 8 Diploma from a vocational academy 9 Bachelor's degree from a university, administration/technical college, vocational academy, diploma from a technical college 10 Master's degree from a university, technical college , or similar, diploma, master's degree, state examination 11 doctorate; habilitation	Summarized into degree categories:<br><br>1 low (1 to 3),<br><br>2 middle (4 to 6),<br><br>3 high school (7-8)<br>4 university (9-11)
**Children in household (children)**	
How many children under the age of 18 live with you at least half the time?<br>open answer field numerical information from 0-99	
**Income and income disclosure (income, income disclosure)**	
If you add up income from all sources (wage, salary, income from self-employment, rental and leasing, pension, pension, government grants, assets, dividends, housing benefit, unemployment benefit, child benefit and other income), what is the total net income of your household (income after deduction of taxes and all duties)? 1 Less than 1,000 euros 2 1,000 to less than 2,000 euros 3 2,000 to less than 3,000 euros 4 3,000 to less than 4,000 euros 5 4,000 to less than 5,000 euros 6 5,000 to less than 7,500 euros 7 7,500 euros or more 8 Don't know 9 No answer	8 and 9 recoded as NA<br><br>New variable added: income

	disclosure = 0 if income is NA (don't know/no answer), otherwise, income disclosure is 1
**Income sufficiency (income sufficiency)**	
What best describes how you assess your current household income? With your current income, you can... 4 living comfortably 3 getting along 2 getting along with difficulty 1 getting along with great difficulty 5 don't know	
**Work, coded full-time vs not (employment)**	
Which term best describes your situation in the last seven days?<br>1 full-time employed (more than 30 hours/week) 2 part-time employed (up to 30 hours/week) 3 marginally employed, 450-euro job, mini-job 4 apprentice/trainee 5 pupil 6 student 7 in retraining 8 currently unemployed 9 currently on short-time work 10 federal voluntary service, voluntary social year (FSJ), voluntary ecological year (FÖJ) 11 in early retirement/early retirement 12 in retirement/pension 13 on maternity leave, parental leave 14 chronically ill or disabled 15 not employed, housework, caring for children or other people	Dummy coded<br>1: full-time employment,<br>0: otherwise
**Leisure time (leisure hours)**	
On a working day (Mon-Fri), how many hours of free time do you have on average when you can do whatever you like? Free time is the time that is left after deducting work, household, studies/training and sleep. open answer field numeric information 0-24	
**Leisure rating (leisure rating)**	
How would you describe your free time situation?<br>7-point scale (1 = too little free time; 7 = too much free time)	
**Social security benefits (social security)**	
Do you receive or have you received basic security from the state in the last 5 years?<br>1 Yes, unemployment benefit I (up to 2 years after unemployment) 2 Yes, unemployment benefit II (for those who are able to work) 3 Yes, social assistance (for those who are not able to work) 4 Yes, housing benefit 5 Yes, something else, namely: [open text field] 6 No, no basic insurance received	Dummy coded:<br>1: Yes, 0: No
**Social class (social class)**	
Today there is a lot of talk about the different social classes. Which class do you classify yourself as?<br>1 lower class 2 working class 3 lower middle class 4 middle middle class 5 upper middle class 6 upper class 7 Don't know	
**Need for cognition (need for cognition)** 7-point Likert scale (1 = does not apply at all; 7 = fully applies).	
I like it when my life is full of tricky tasks that I have to solve.<br>I prefer complicated problems to simple problems.	Mean score
**New media literacy scale (new media literacy)**	
To what extent do you agree with each statement? (1-7 Likert scale)<br><br>I find it easy to create user accounts and profiles on the Internet.	Mean score

I can use the software necessary for the creation of media content (text, image, video, etc.).<br><br>I am good at sharing digital media content and news on the internet.<br><br>I may post or comment on media content shared by others.<br><br>I know how to use search engines and research tools to get the information I need.<br>I find it easy to use a wide variety of media and sources to obtain information.	
**Residence type (residence urban, residence suburban, residence rural)**	
Numeric area code field, coded for urban, suburban and rural provided by panel	Dummy coded rural: yes (0), no (1);<br><br>suburban yes (0), no(1);<br>urban: yes (0), no(1);
**What state do you live in? (east german)**	
Text field: auto-complete with German states	Dummy coded: 0 west, 1 east
**Civic Skill scales (writing skills, presentation skills, organisation skills, meeting skills)**	
In which contexts have you carried out one of the following activities in the last 6 months?<br><br>Answer options: 1 in the work context 2 in the private context 3 in the context of membership in political organisations 4 not performed at all in the above contexts<br><br>Presentation skill: gave a presentation or speech<br><br>Meeting skill: attended a meeting where decisions were made<br><br>Organization skill: scheduled or chaired a meeting<br>Writing skills: wrote a letter or email	Sum of answer options: 1,2,3<br>If 4 selected: coded 0
**Political leaning (pol. leaning)**	
In politics one sometimes speaks of “left” and “right”. Where on the scale would you place yourself if 0 is left and 10 is right? 11 point scale (0 = left; 10 = right)	Recoded to 11 point scale (1 = left; 11 = right)
**Political interest (pol. interest)**	
How strongly are you interested in political issues? Likert points (5 = very strongly, 4 = strongly, 3 = moderately, 2 = little or 1 = not at all).	
**Political debate online and personal (pol. debate online, pol. debate offline)**	
On how many days in the past week have you…	

... discussed personally with other people, e.g. family members, friends or acquaintances, about political issues?<br><br>... discuss political issues with other people in online forums or on other Internet channels, e.g. messenger services or social media?<br>1 not at all 2 1 day 3 2 days 4 3 days 5 4 days 6 5 days 7 6 days 8 7 days	
**Political knowledge system (political knowledge)**	
- By whom is the Federal Chancellor of the Federal Republic (Germany) elected?<br>1 By the people 2 By the Bundesrat 3 By the Bundestag 4 By the Federal Assembly 5 Don't know<br><br>- What does the term "voting secrecy" mean?<br>1 You mustn't talk about who you voted for 2 You don't find out which candidate you voted for 3 You can only vote for one party 4 Nobody can find out who you voted for unless you tell them 5 Don't know<br><br>- In which elections can EU citizens living in Germany vote even if they do not have German citizenship?<br>1 In federal elections 2 In local elections 3 In state elections 4 Not allowed to vote in any of these 5 Don't know<br><br>- Who has the so-called guideline authority?<br>1 The Foreign Minister 2 The Federal Chancellor 3 The Federal President 4 The President of the Bundestag 5 Don't know<br><br>- Compare only the parties specified here. Which party currently has the most seats in the German Bundestag?<br>1 FDP 2 AfD (Alternative for Germany) 3 Die Linke 4 Bündnis90/Die Grünen 5 Don't know<br>- The solidarity surcharge is a surcharge on income and corporation tax. What is it for?<br>1 To finance German mining 2 To finance German unity 3 To finance statutory pensions 4 To finance the reform of the healthcare system 5 Don't know	Mean score of correct answers
**Political knowledge politicians (politician knowledge)**	
Please assign these politicians to your parties. 1 CDU 2 FDP 3 SPD 4 Greens 5 AfD 6 Don't know<br>1. Markus Soeder 2. Ursula von der Leyen 3. Nicola Beer 4. Christian Lindner 5. Andrea Nahles 6. Olaf Scholz 7. Heiko Maas 8.Robert Habeck 9. Annalena Baerbock 10. Stephan Brandner 11.Christine Lambrecht	Mean score of correct answers
**Political self-efficacy (pol. efficacy)**	
I can understand and assess important political issues well.<br><br>I trust myself to take an active part in a conversation about political issues.<br>7-point Likert scale (1 = does not apply at all; 7 = fully applies).	Mean score
**News use online (news use online)**	
How many days a week do you on average consume political news on the internet (all devices)? Please include all, also news websites, video sites, online offers from newspapers and magazines.<br>1. less than one day a week 2. one day a week 3. on 2 days a week 4. on 3 days a week 5. on 4 days a week 6. on 5 days a week 7. on 6 days a week 8. on 7 days a week 9 I don't use news on the internet	Recoded 9 = 0

**Political participation offline (pol. online action)**	
Have you done any of the following in the last 12 months? Have you …<br><br>1 Contacted a federal or local political figure? yes/no<br><br>2 Worked informally with others on community issues? yes/no<br><br>3 Participated in a protest action? yes/no<br><br>4 Attended a regular political meeting or election campaign? yes/no<br>5 Boycotted certain products/brands? yes/no	Mean score of binary (0…1)
**Political participation online (pol. offline action)**	
Have you done any of the following in the last 12 months? Have you …<br><br>1. Posted, tweeted or sent your own political contributions to social media via mailing lists? yes/no<br><br>2. Forwarded or shared other people's political posts on social media? yes/no<br><br>3. Composed comments on political contributions, articles or programs? yes/no<br><br>4. Written articles on political issues for a blog? yes/no<br>5. Used public participation platforms used by government agencies? yes/no	Mean score of binary (0…1)
**Election voted (voted in election)**	
Did you vote in the national election 2021? yes/no	1: yes, 0: no
**Membership organizations (religious membership, union membership,)**	
Are you a member of one of the following organisations, or are you actively involved in them? 1 political party 2 querdenker movement 3 energy community 4 work union 5 religious or church group 6 employer organisation 7 environmental organisation 8 other political organisation 9 humanitarian group 10 peace activist group 11 others 12 none	Dummy coded for 4 (1: yes; 0:no) and 5 (1: yes; 0:no)
**Religiousness (religiousness)**	
How religious are you? 7-point Likert scale (1 = not at all religious; 7 = very religious)	
**Social Media Usage (SM Youtube, SM Whatsapp, SM Twitter, SM Telegram,S M Signal, SM Instagram, SM Facebook, SM variety)**	
Please indicate which of these social media apps you use.<br><br>1 Discord 2 Facebook 3 Instagram 4 LinkedIn 5 Nebenan.de 6 Pinterest 7 Reddit 8 Signal 9 Snapchat 10 TikTok 11 Telegram 12 Threema 13 Tumblr 14 Twitter 15 Twitch 16 WhatsApp 17 XING 18 Youtube 19 Other, namely [open answer field] 20 I use no social media	**Selection:** yes (1)/no (0)<br>**Variety:** count of selected

S1.2. Survey items – full correlation table

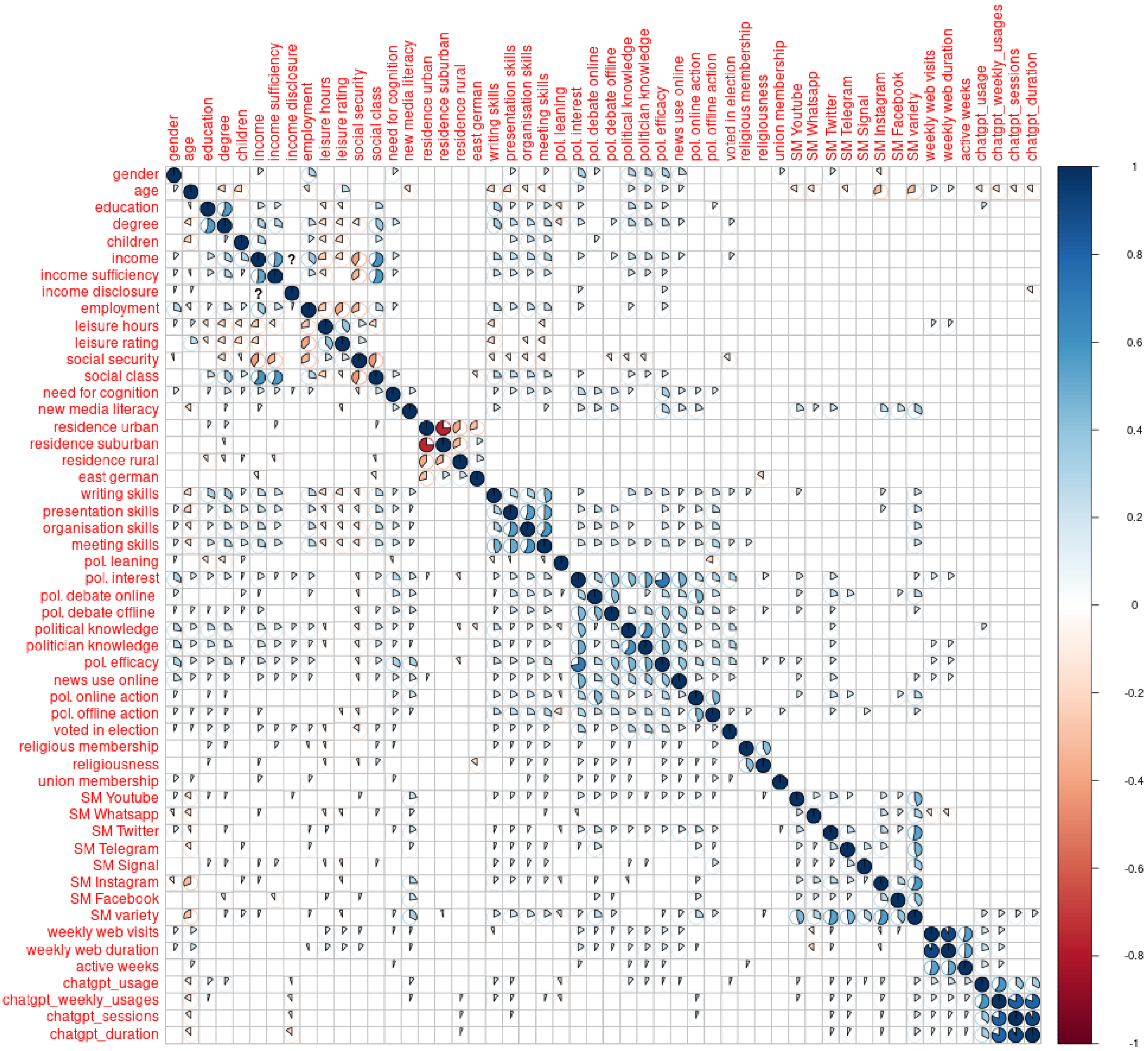

S1.2 Fig. Top right triangle shows Bonferroni-corrected correlations, bottom left triangle uncorrected correlations.

S2. Demographics distribution of sample (age, gender, education crossed).

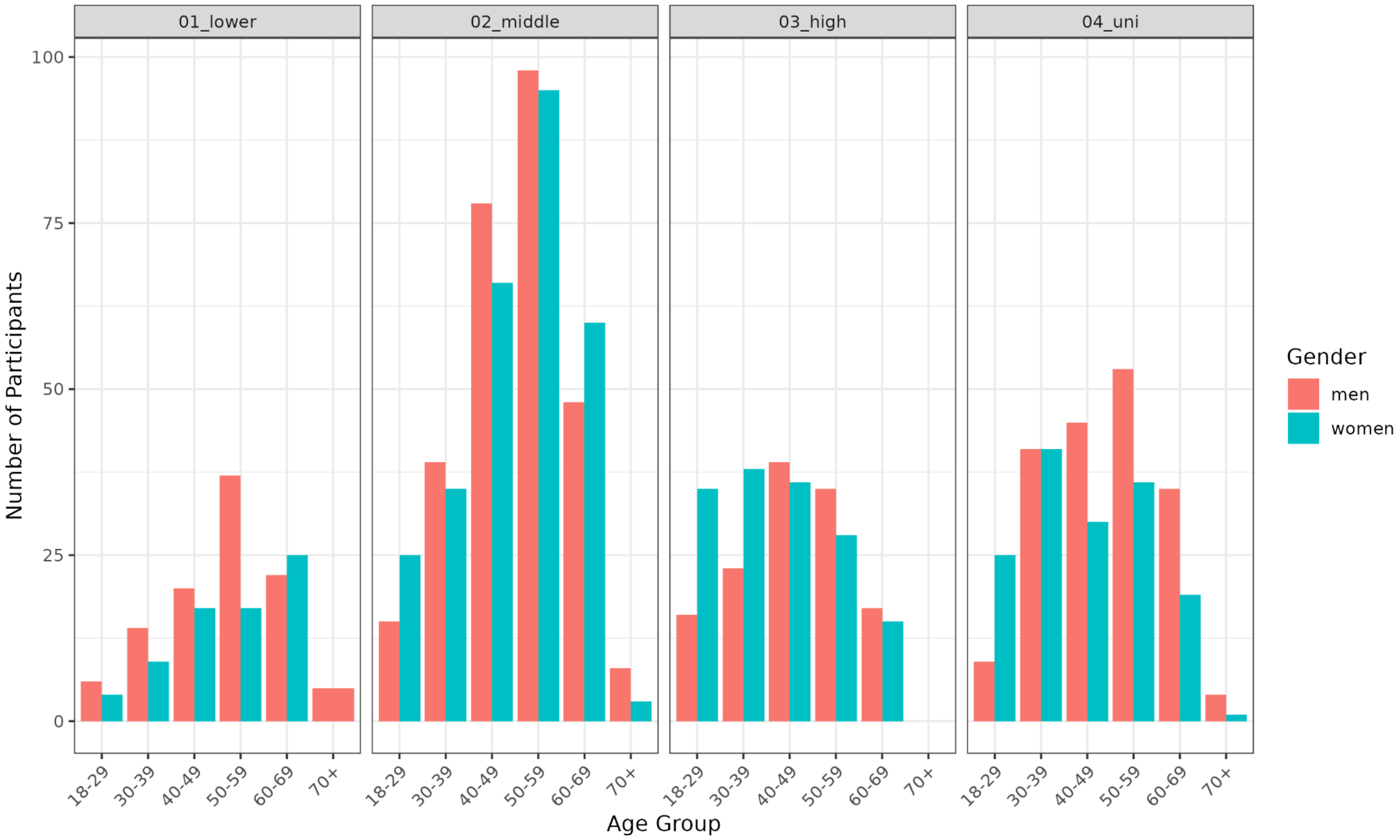

S2 Fig. Percentage of participants in our sample across gender, ages and education levels, crossed.

S3 Web-tracking information

S3.1 Text Domain visits explained

For analyses, from the web browsing data, we used three response variables to measure participant’s ChatGPT activity, i.e. activity on chat.openai.com: ChatGPT usage (dummy coded, 1 = visited chat.openai.com at least once); and engagement: chat.openai.com visits (log-transformed), chat.openai.com visit duration (sum of all visit durations, log-transformed). We also used participants' overall web activity (domain visits excluding chat.openai.com) as an independent variable.

Visits here are aggregated sequences of pages belonging to the same domain loaded within 30 minutes of each other. This aggregation reduces biases due to (1) high number of page loads when registering, logging or re-loading ChatGPT (a common occurrence during the months of highest demand and short supply, see Fig S3) and, in general, (2) standardizing the diverse browsing patterns that different domains demands from their visitors, as some require more page loads than others.

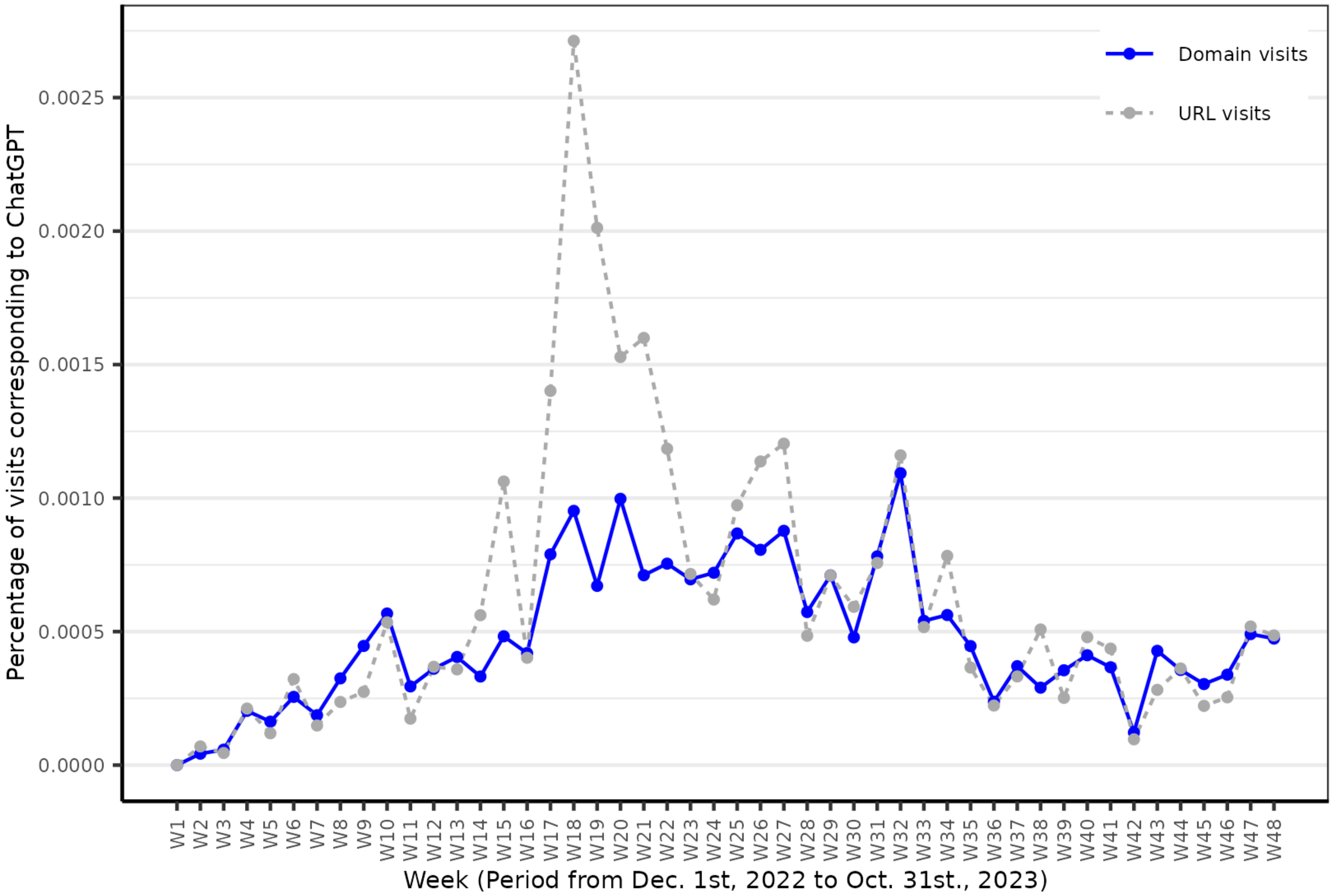

S3.1 Fig. Percentage of ChatGPT Domain and URL visits. The X-axis indicates the week starting from Dec 1st., 2022. The Y-axis indicates the percentage of ChatGPT visits. The legend indicates if the visits are calculated based on the URL or the domain (aggregating sequences of URLs belonging to chat.openai.com loaded within 30 minutes of each other).

S3.2 ChatGPT visits and adoption Distribution

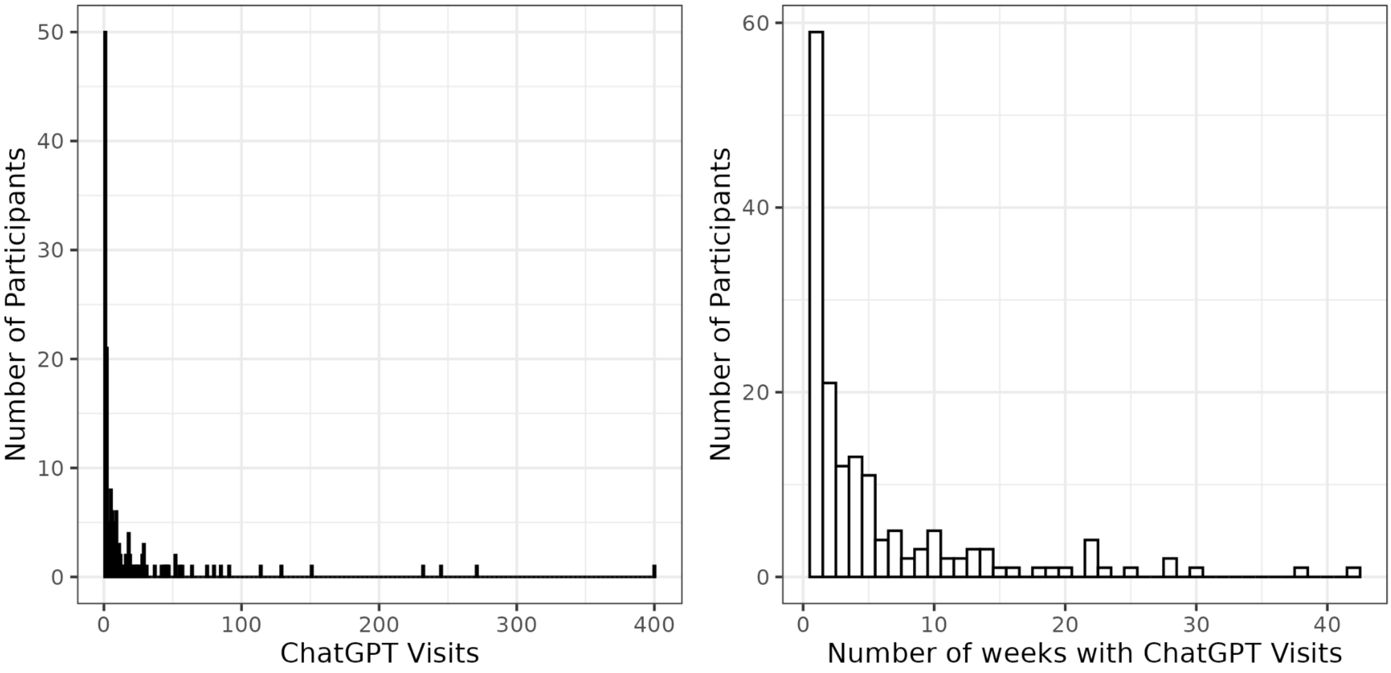

S3.2 Fig. Distribution of ChatGPT Visits (left) and Adoption (right).

S4. Non-imputed regressions

S4.1 Table. Regressions tables of the non-imputed data.

	Usage			Visits			Adoption		
Predictors	*Odds Ratios*	*CI*	*p*	*Incidence Rate Ratios*	*CI*	*p*	*Incidence Rate Ratios*	*CI*	*p*
(Intercept)	0.05	0.04 – 0.07	**<0.001**	5.34	3.88 – 7.50	**<0.001**	2.34	1.75 – 3.13	**<0.001**
gender	1.13	0.92 – 1.39	0.258	1.11	0.84 – 1.47	0.358	1.01	0.86 – 1.19	0.875
age	0.53	0.43 – 0.66	**<0.001**	0.72	0.58 – 0.89	**0.002**	0.82	0.71 – 0.95	**0.012**
education	1.24	1.02 – 1.49	**0.026**	0.85	0.69 – 1.05	0.069	0.94	0.83 – 1.08	0.391
income	1.06	0.86 – 1.31	0.567	0.86	0.68 – 1.08	0.171	0.91	0.78 – 1.06	0.237
residence rural	1.22	1.01 – 1.46	**0.038**	1.03	0.85 – 1.28	0.749	1.06	0.92 – 1.22	0.407
children	0.84	0.68 – 1.03	0.098						
employment	0.82	0.66 – 1.01	0.064						
new media literacy	1.23	0.98 – 1.56	0.077						
pol leaning	0.83	0.68 – 1.01	0.060						
pol debate online	0.81	0.66 – 0.99	**0.044**						
political knowledge	1.45	1.15 – 1.83	**0.002**						
voted in election	1.36	1.03 – 1.92	0.051						
SM Telegram	1.09	0.89 – 1.33	0.392						
SM Facebook	0.79	0.64 – 0.98	**0.029**						
SM variety	1.40	1.11 – 1.77	**0.005**	1.20	0.97 – 1.48	0.080	1.12	0.97 – 1.29	0.139
weekly web duration	2.52	1.87 – 3.42	**<0.001**						
active weeks	2.32	1.65 – 3.44	**<0.001**				1.44	0.98 – 2.10	**0.047**
need for cognition				1.37	1.06 – 1.78	**0.007**	1.21	1.02 – 1.45	**0.027**
writing skills				1.56	1.18 – 2.05	**<0.001**	1.34	1.10 – 1.63	**0.003**
presentation skills				1.13	0.91 – 1.42	0.242			
pol efficacy				0.76	0.54 – 1.07	**0.046**			
pol online action				1.23	1.00 – 1.54	**0.038**	1.24	1.06 – 1.46	**0.003**
religious membership				0.88	0.72 – 1.11	0.211	0.85	0.73 – 0.99	**0.034**
SM Whatsapp				1.29	0.97 – 1.69	**0.047**			
weekly web visits				2.14	1.48 – 3.08	**<0.001**	1.41	1.11 – 1.80	**0.008**
organisation skills							0.74	0.61 – 0.89	**0.001**
meeting skills							1.25	1.05 – 1.51	**0.014**
pol debate offline							0.90	0.77 – 1.06	0.185
Observations	1319			145			146		
R^2 Tjur	0.198			0.629			0.585		
AIC	759.353			1034.112			771.395		
AICc	759.880			1038.362			776.176		

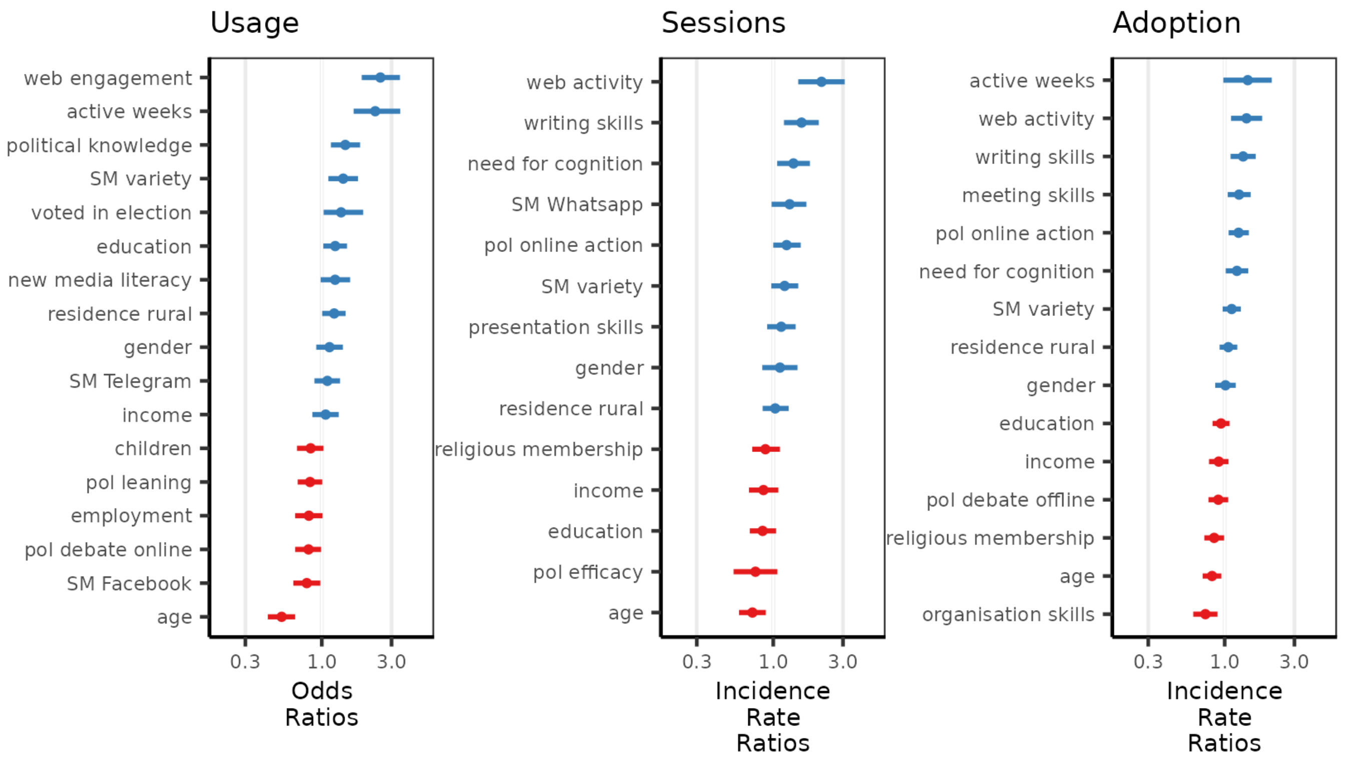

S4.2 Fig. Odds and incidence rate ratios estimates of the regression on the non-imputed data.

S5. Feature Selection

S5.1. Histograms of iteration in which features are selected by LASSO

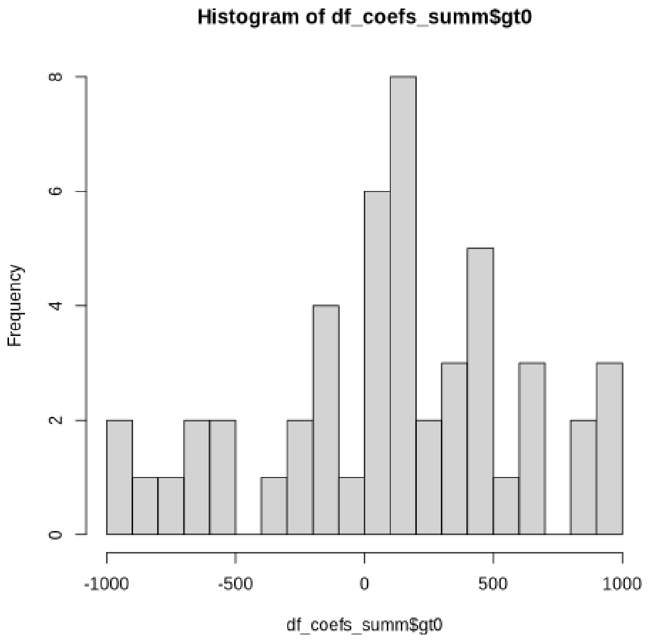

S5.1.1 Fig. Histogram of interactions in which features are selected for ChatGPT Usage.

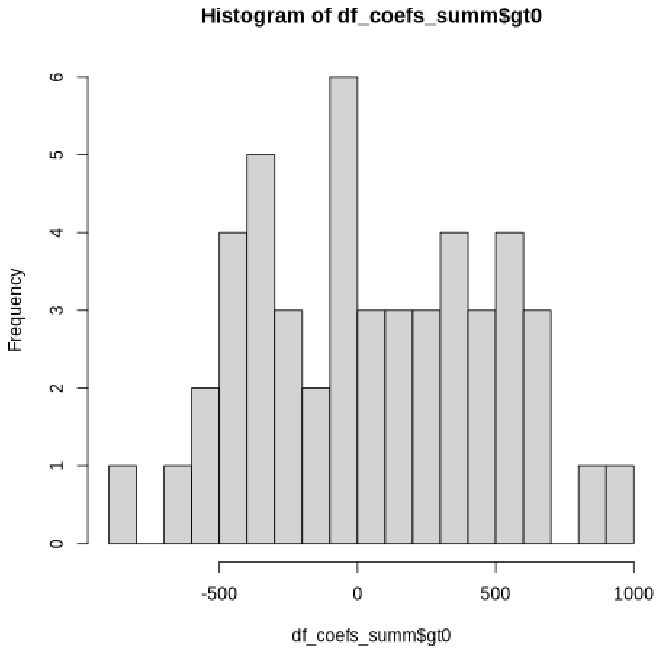

S5.1.2 Fig. Histogram of interactions in which features are selected for ChatGPT Visits.

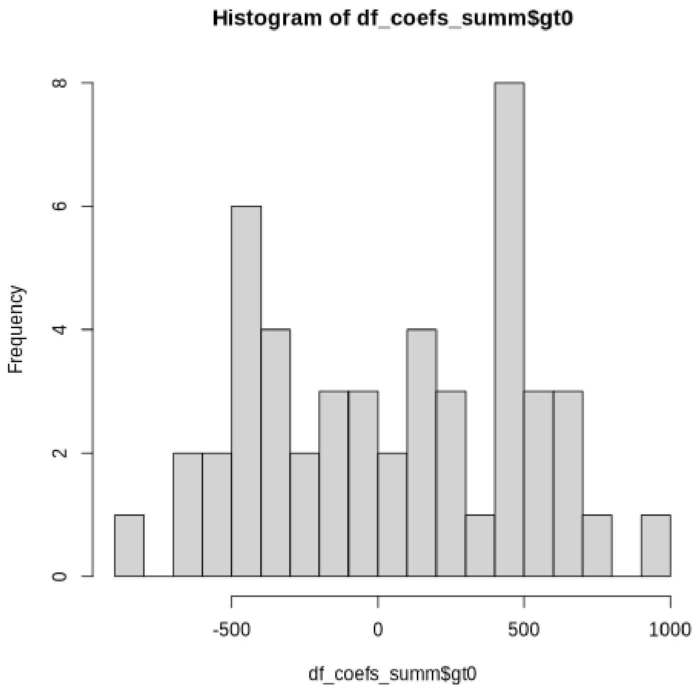

S5.1.3 Fig. Histogram of interactions in which features are selected for ChatGPT Adoption.

S5.2. Examples of cross-validation curves in LASSO iterations

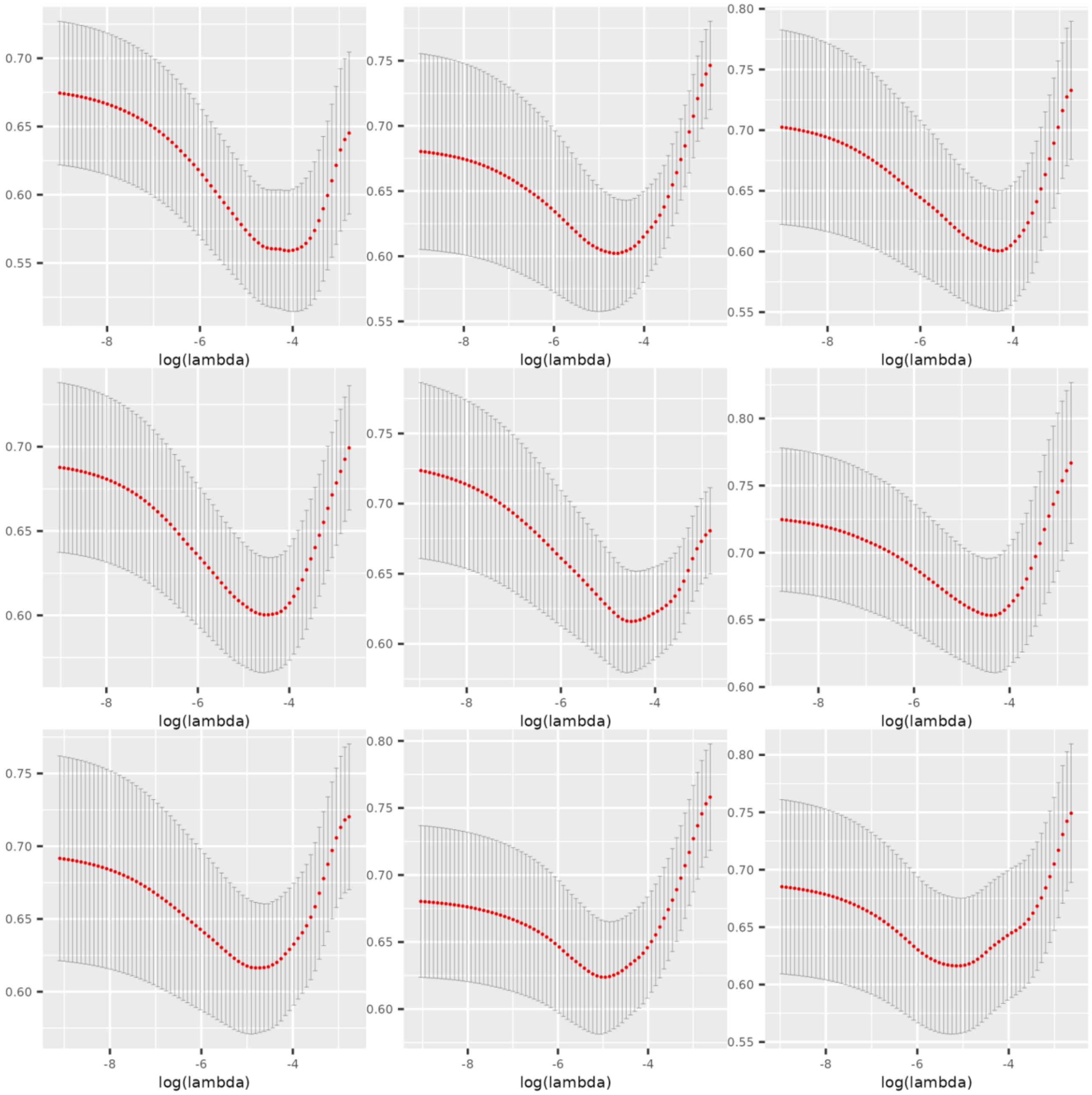

S5.2.1 Fig. Cross-validation curves for ChatGPT Usage.

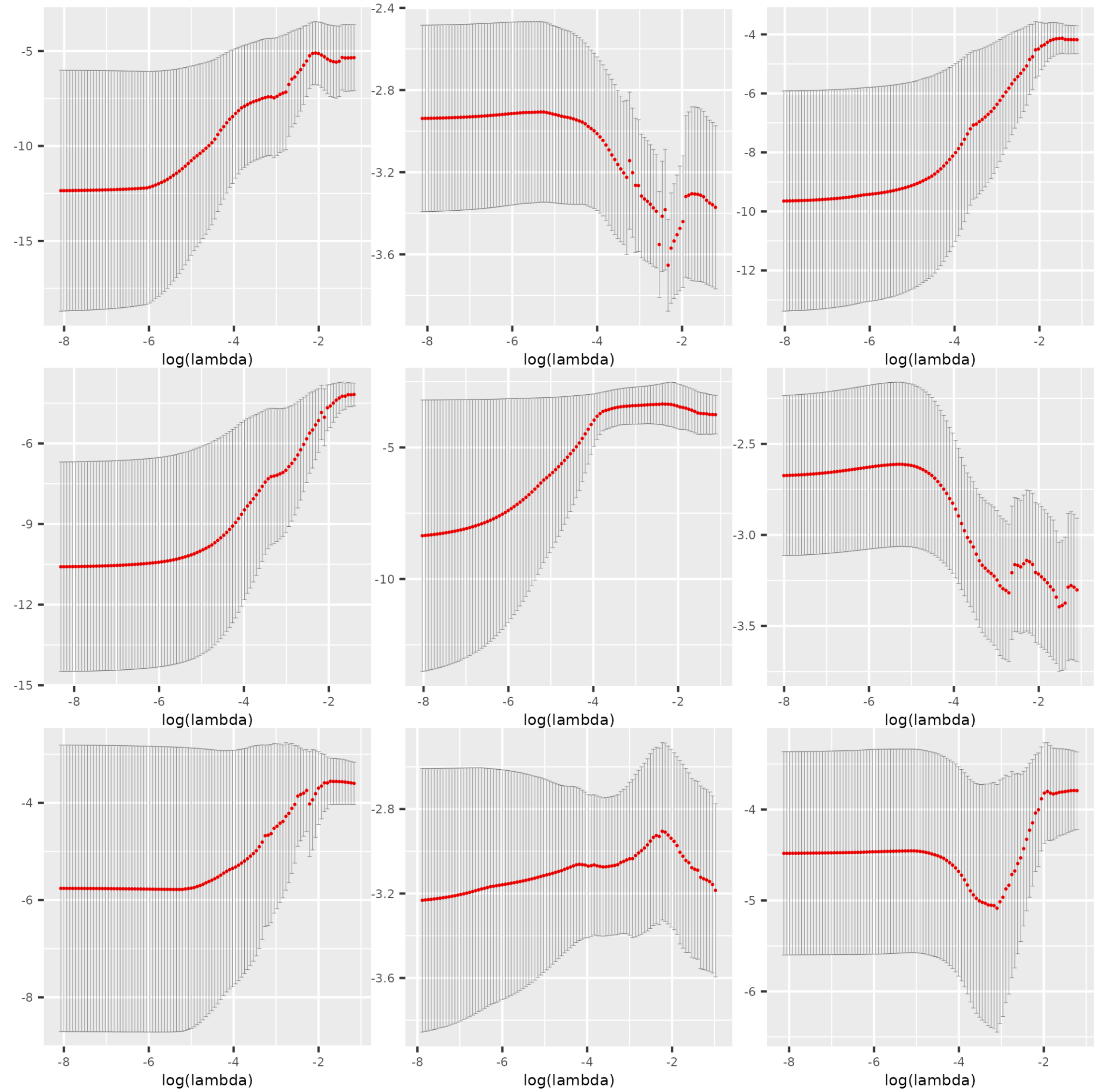

S5.2.2 Fig. Cross-validation curves for ChatGPT Visits

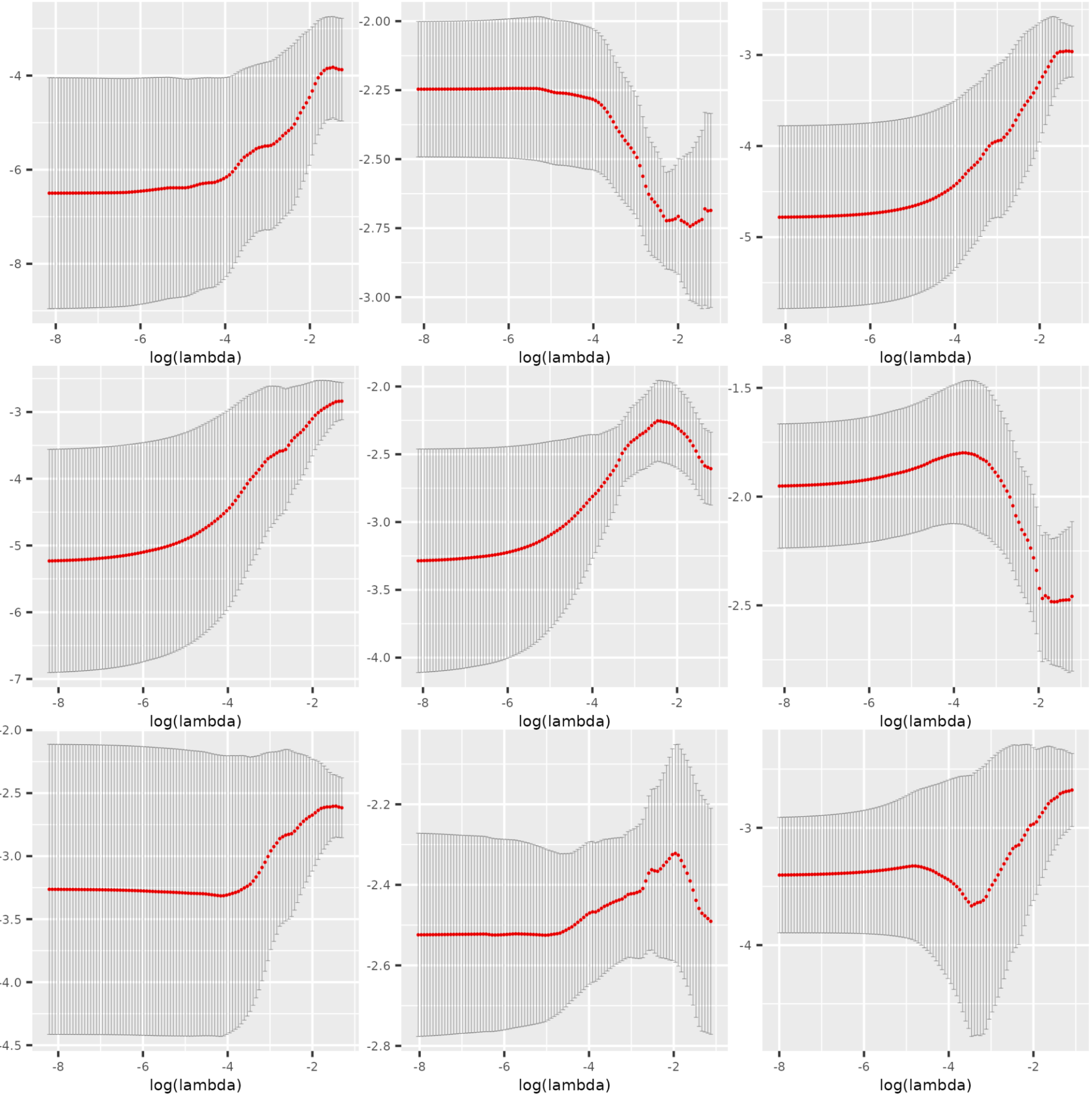

S5.2.3 Fig. Cross-validation curves for ChatGPT Adoption.

S5.3. Examples of coefficient plots in LASSO iterations

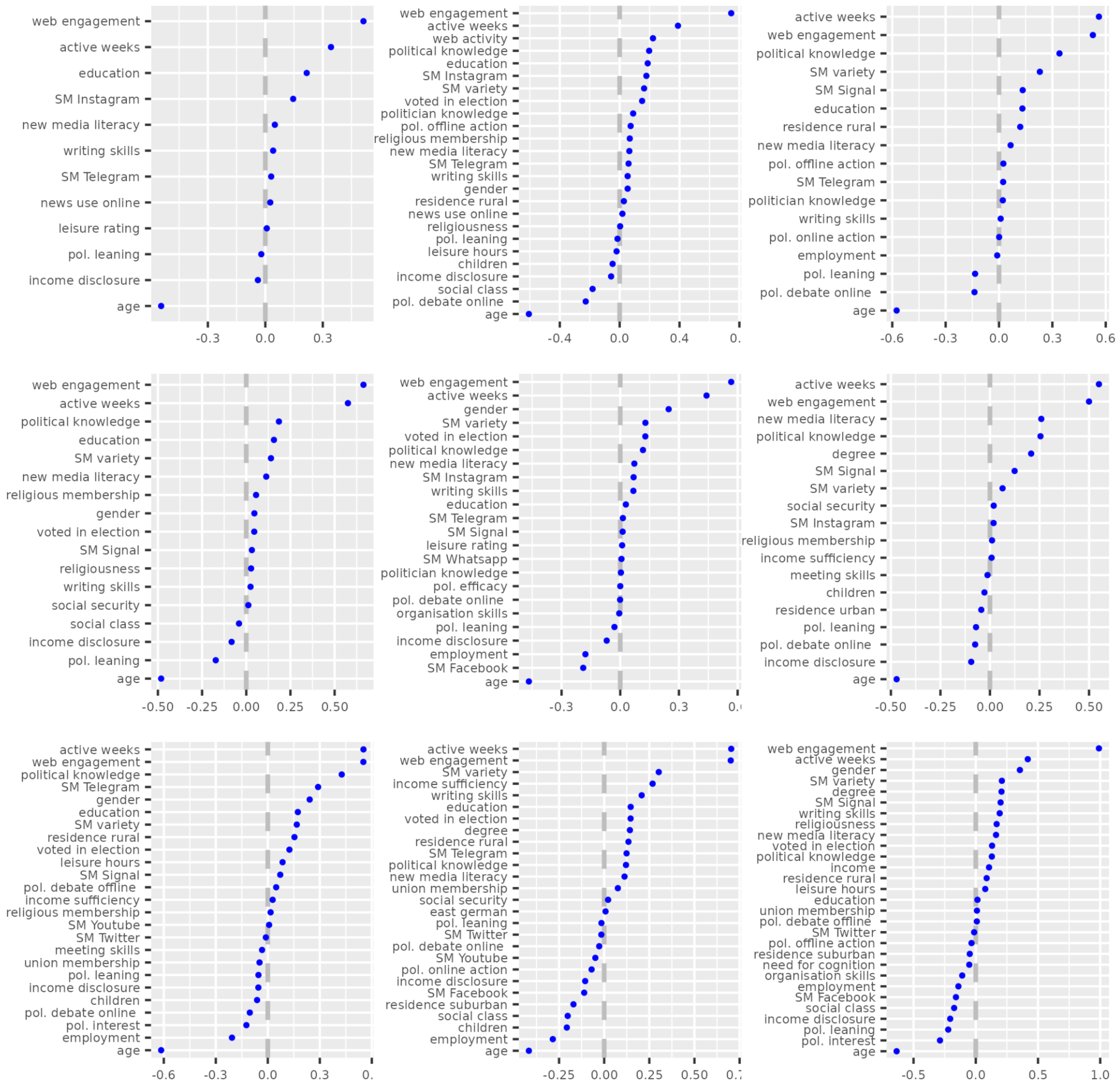

S5.3.1 Fig. Coefficient plots for ChatGPT Usage.

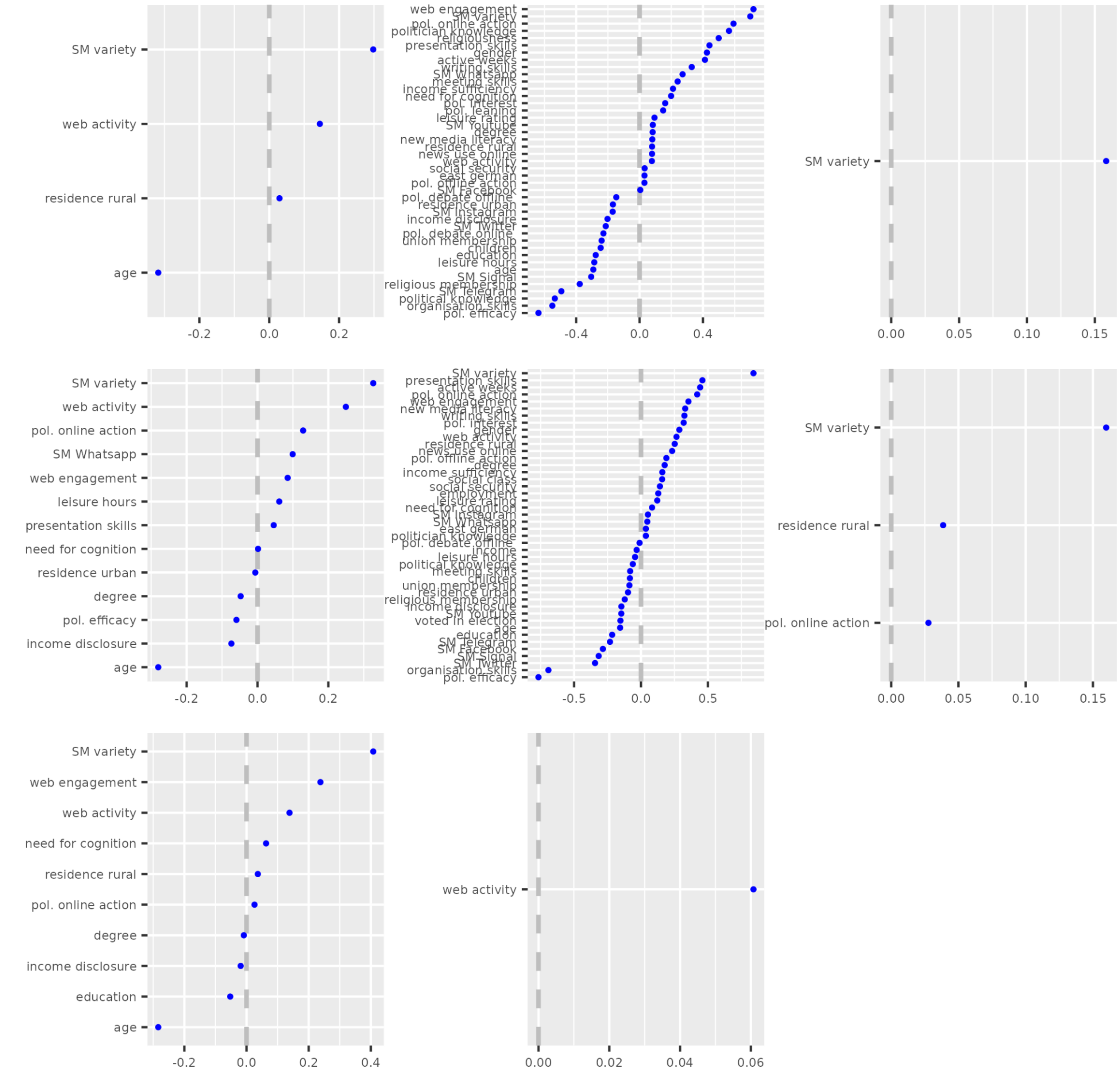

S5.3.2 Fig. Coefficient plots for ChatGPT Visits.

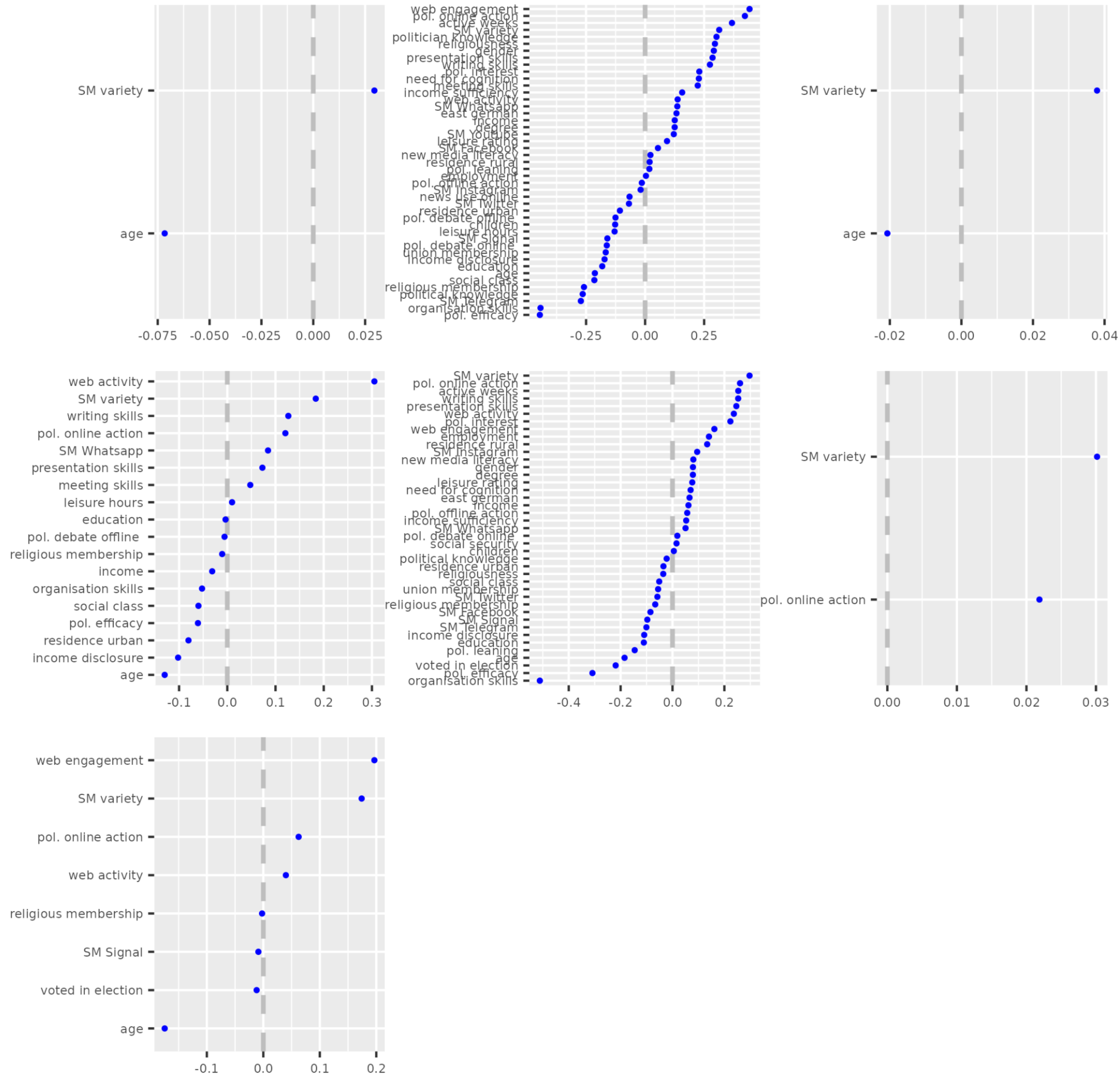

S5.3.3 Fig. Coefficient plots for ChatGPT Adoption.

S6. Examples of Receiver Operating Characteristic (ROC) curves for ChatGPT Usage

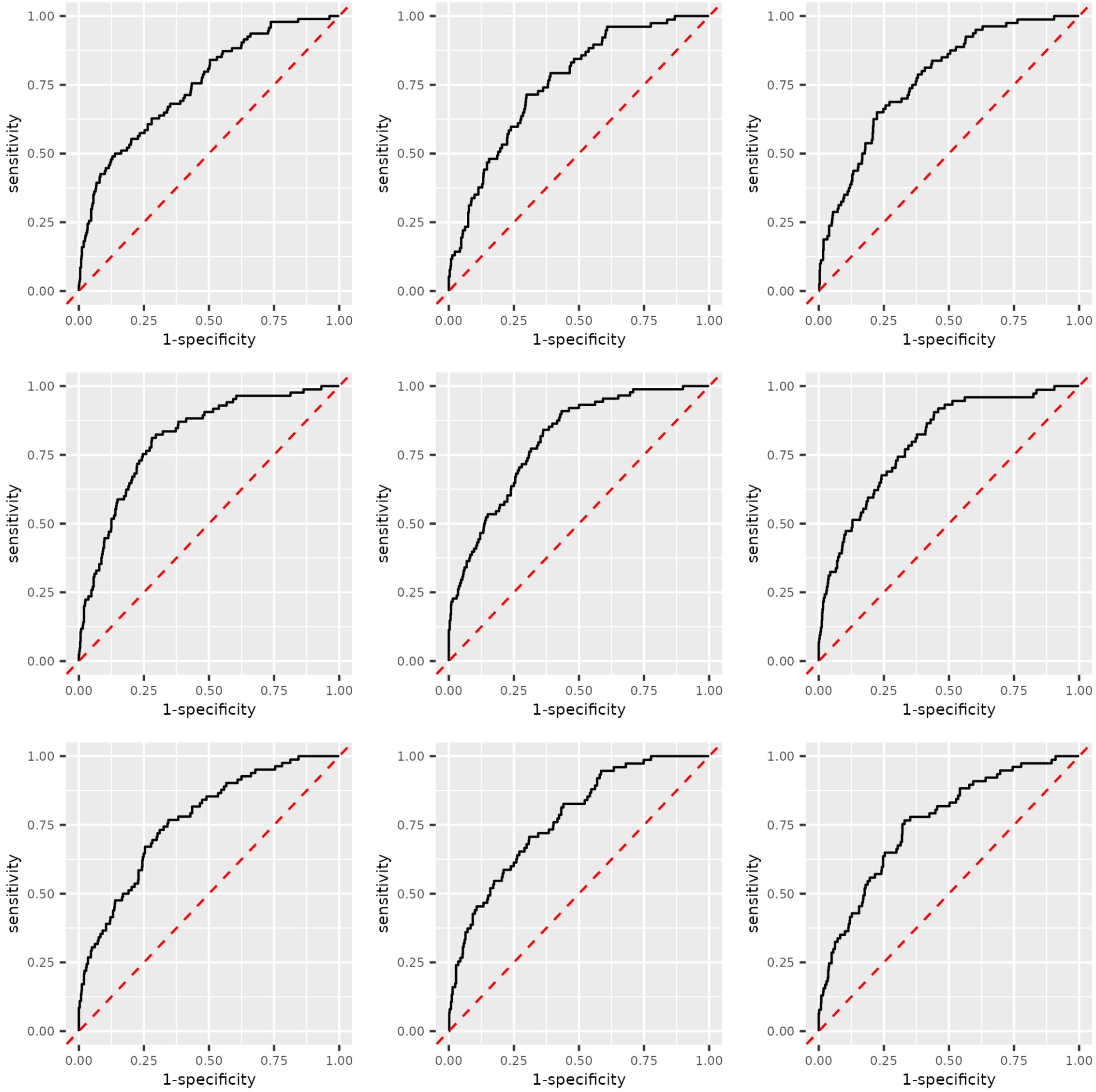

S6.1 Fig. Receiver Operating Characteristic (ROC) curves for ChatGPT Usage (incl. web activity feature)

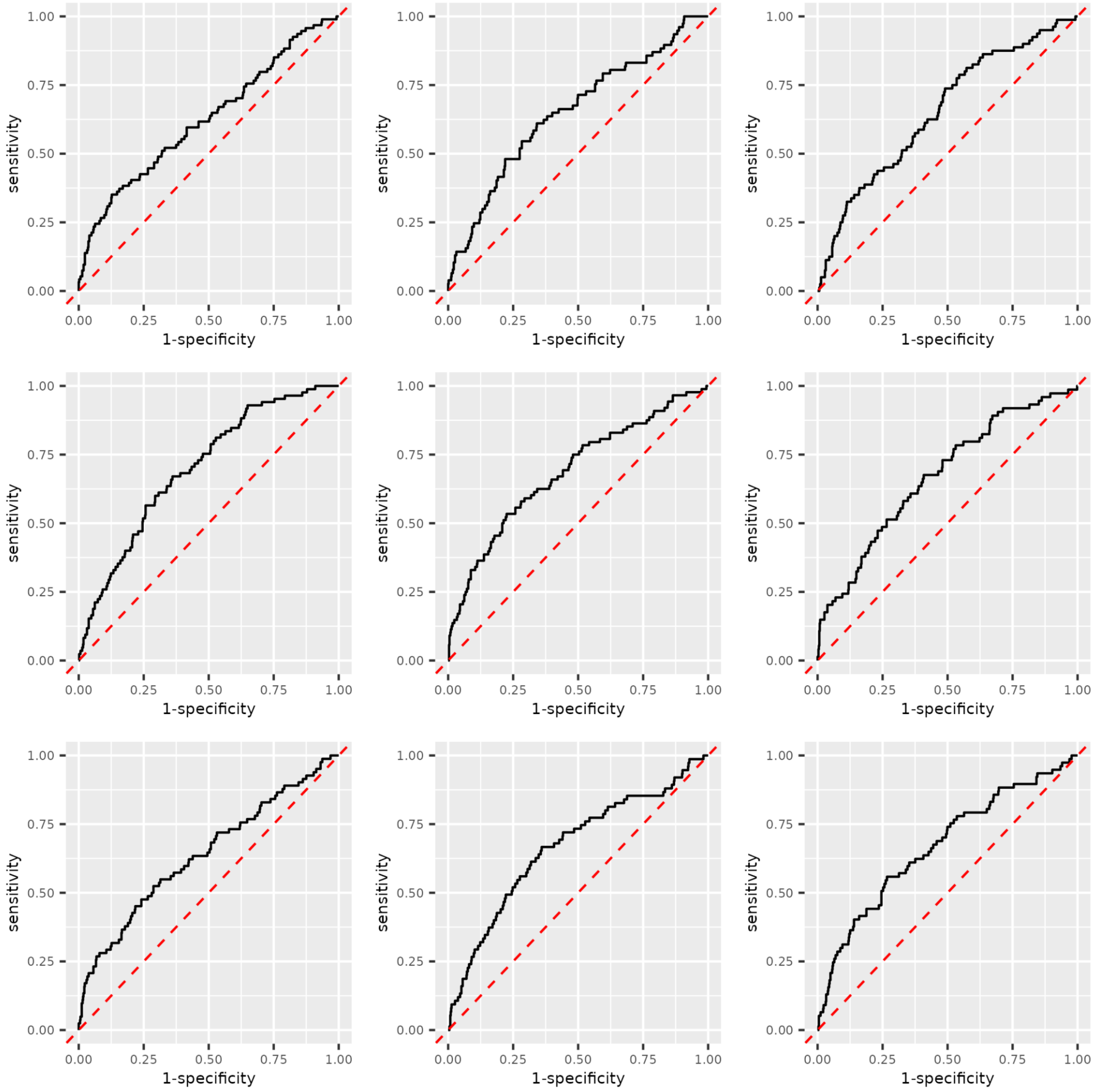

S6.2 Fig. Receiver Operating Characteristic (ROC) curves for ChatGPT Usage (excl. web activity feature)

S7. Data checks

S7.1 Correlations Tables

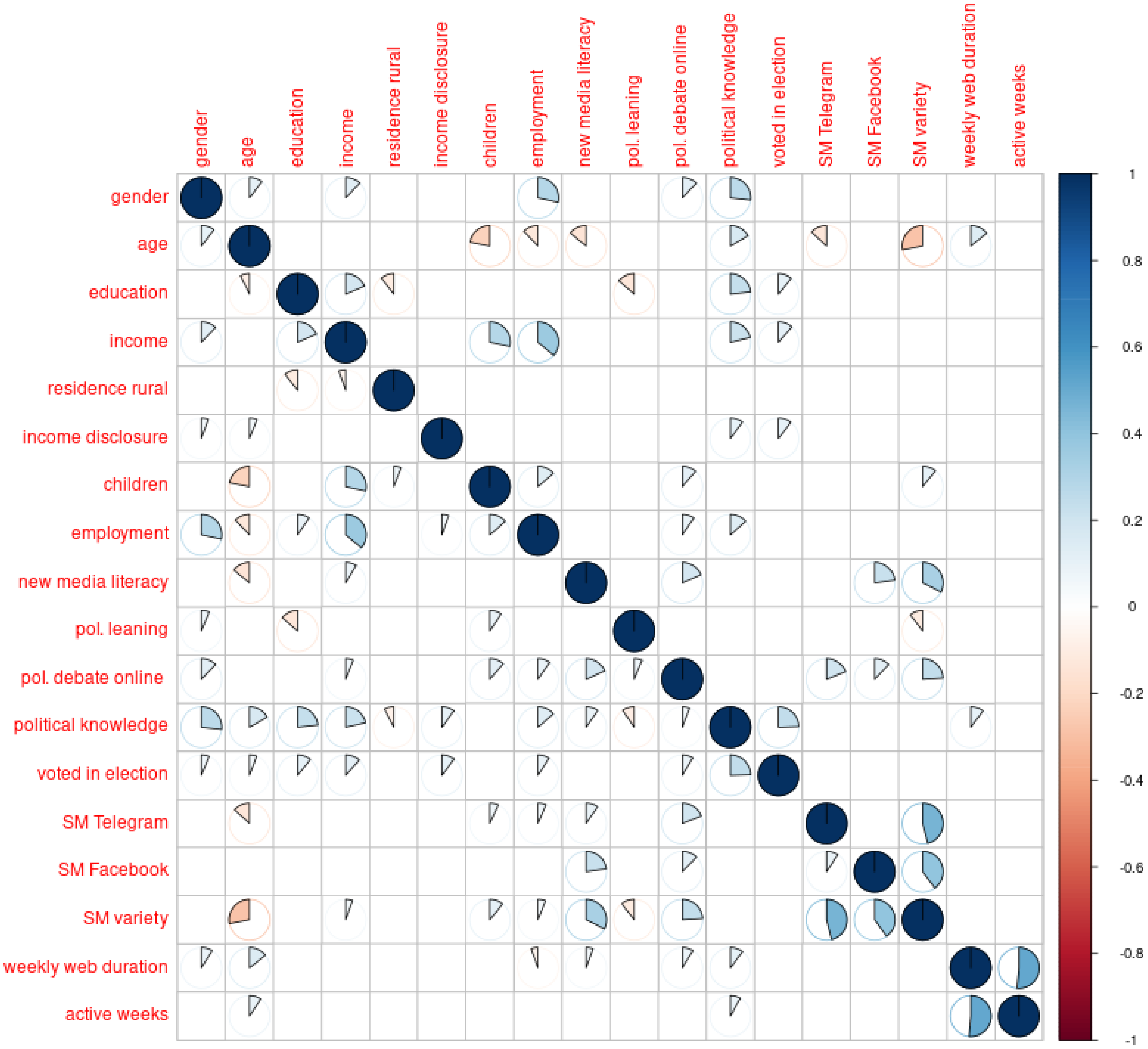

S7.1.1 Fig. Correlation of (LASSO) selected variables for ChatGPT Usage.

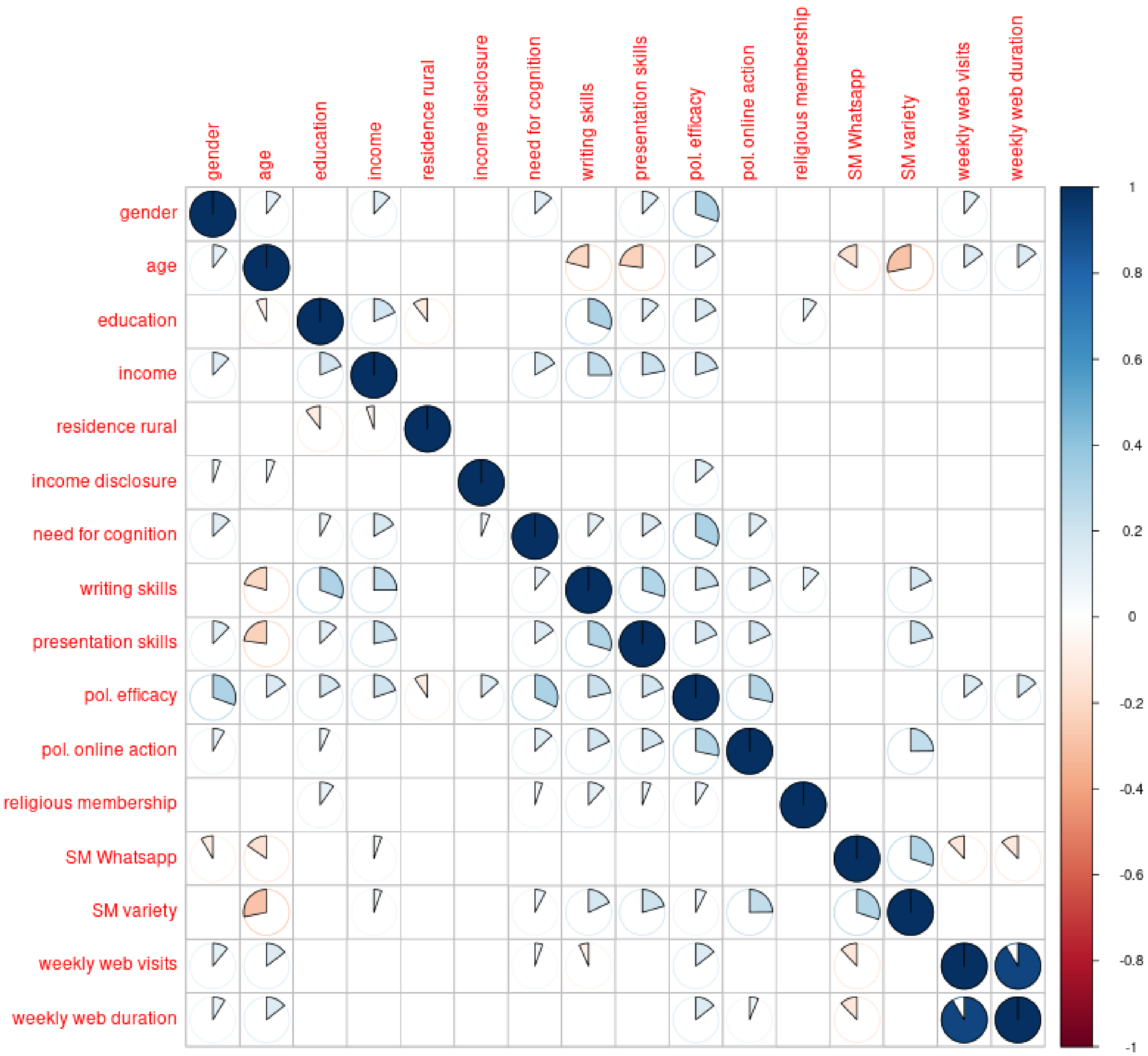

S7.1.2 Fig. Correlation of (LASSO) selected variables for ChatGPT Visits.

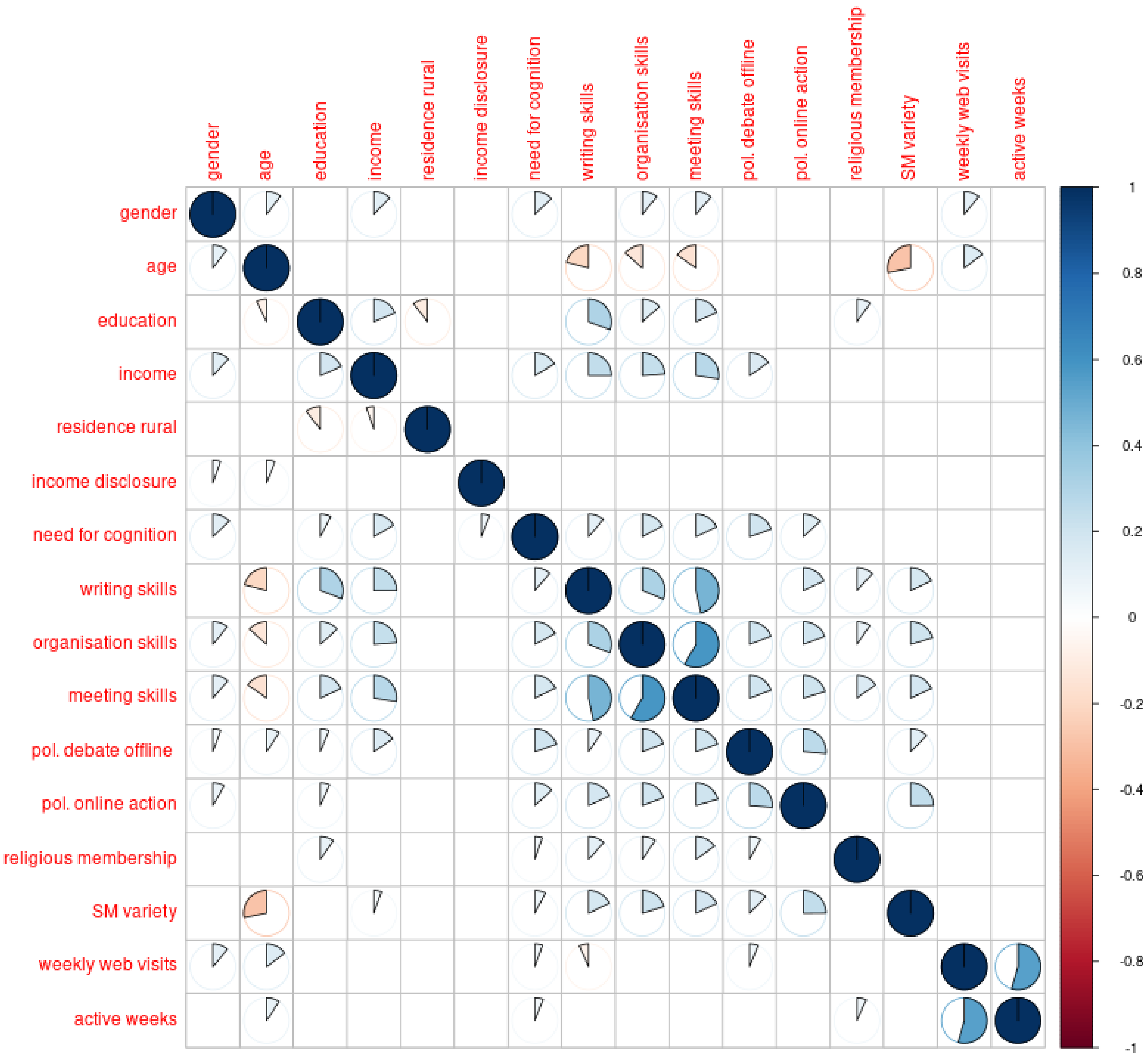

S7.1.3 Fig. Correlation of (LASSO) selected variables for ChatGPT Adoption.

S7.2 Variable Inflation Factor

S7.2. Table. Overview over survey constructs used in the lasso regression feature selection.

feature	Usage	Visits	Adoption
SM variety	1.76	1.18	1.196
income	1.377	1.302	1.239
SM Telegram	1.311	-	-
employment	1.304	-	-
SM Facebook	1.291	-	-
age	1.281	1.453	1.362
political knowledge	1.234	-	-
gender	1.214	1.202	1.082
weekly web duration	1.162	-	-
pol. debate online	1.158	-	-
children	1.152	-	-
new media literacy	1.133	-	-
education	1.121	1.125	1.106
pol. leaning	1.088	-	-
active weeks	1.081	-	1.139
voted in election	1.062	-	-
residence rural	1.037	1.161	1.114
pol. efficacy	-	1.906	-
weekly web visits	-	1.36	1.317
writing skills	-	1.311	1.423
presentation skills	-	1.251	-
SM Whatsapp	-	1.231	-
pol. online action	-	1.184	1.263
income disclosure	-	1.169	1.161
religious membership	-	1.148	1.264
need for cognition	-	1.132	1.159
meeting skills	-	-	1.784
organisation skills	-	-	1.71
pol. debate offline	-	-	1.236

VIF values of imputed models. For a model of visits that includes both web engagement and web activity, the VIF values are 4.11 and 4.23, respectively.

S8. Zero-Inflation Models

S8.1 Table. Hurdle model with a negative binomial and zero-inflation negative binomial model for ChatGPT Visits.

	**Hurdle (NB)**			**ZINB**		
Predictors	*Incidence Rate Ratios*	*CI*	*p*	*Incidence Rate Ratios*	*CI*	*p*
(Intercept)	0.99	0.14 – 6.76	0.988	2.51	1.52 – 4.15	**<0.001**
gender	1.35	0.84 – 2.18	0.210	1.27	0.89 – 1.80	0.185
age	0.71	0.51 – 0.98	**0.040**	0.69	0.53 – 0.90	**0.005**
education	0.84	0.59 – 1.22	0.363	0.91	0.69 – 1.21	0.518
income	0.95	0.66 – 1.37	0.776	1.01	0.74 – 1.38	0.939
residence rural	0.98	0.69 – 1.39	0.911	0.98	0.75 – 1.29	0.892
income disclosure	0.79	0.56 – 1.10	0.166	0.80	0.62 – 1.04	0.101
need for cognition	1.51	0.97 – 2.37	0.070	1.44	1.04 – 1.99	**0.026**
writing skills	1.98	1.25 – 3.14	**0.004**	1.64	1.19 – 2.26	**0.002**
presentation skills	1.25	0.85 – 1.84	0.254	1.26	0.93 – 1.69	0.134
pol efficacy	0.48	0.25 – 0.92	**0.028**	0.57	0.37 – 0.89	**0.014**
pol online action	1.33	0.91 – 1.93	0.139	1.29	0.98 – 1.70	0.071
religious membership	0.90	0.63 – 1.28	0.545	0.95	0.73 – 1.24	0.727
SM Whatsapp	1.36	0.93 – 1.98	0.110	1.27	0.96 – 1.68	0.100
SM variety	1.49	1.03 – 2.15	**0.036**	1.50	1.13 – 1.99	**0.005**
weekly web visits	2.56	1.40 – 4.67	**0.002**	2.57	1.59 – 4.16	**<0.001**
**Zero-Inflated Model**						
(Intercept)	0.05	0.04 – 0.07	**<0.001**	7.86	4.75 – 13.03	**<0.001**
gender	1.17	0.95 – 1.44	0.144	0.87	0.66 – 1.16	0.344
age	0.51	0.41 – 0.63	**<0.001**	1.82	1.38 – 2.42	**<0.001**
education	1.23	1.02 – 1.48	**0.028**	0.77	0.60 – 1.00	**0.048**
income	1.09	0.89 – 1.34	0.384	0.97	0.72 – 1.30	0.850
residence rural	1.19	1.00 – 1.43	0.054	0.80	0.62 – 1.05	0.105
income disclosure	0.79	0.67 – 0.94	**0.007**	1.22	0.97 – 1.54	0.088
children	0.81	0.66 – 0.99	**0.043**	1.30	1.00 – 1.69	**0.046**
employment	0.80	0.65 – 0.99	**0.042**	1.28	0.98 – 1.67	0.073
new media literacy	1.24	0.98 – 1.56	0.069	0.77	0.57 – 1.02	0.070
pol leaning	0.84	0.69 – 1.01	0.065	1.22	0.95 – 1.57	0.120
pol debate online	0.82	0.67 – 1.00	**0.047**	1.36	1.05 – 1.74	**0.018**
political knowledge	1.41	1.13 – 1.76	**0.002**	0.65	0.48 – 0.88	**0.005**
voted in election	1.32	1.01 – 1.72	**0.042**	0.74	0.55 – 1.01	0.061
SM Telegram	1.14	0.94 – 1.38	0.186	0.84	0.65 – 1.09	0.197
SM Facebook	0.80	0.65 – 0.98	**0.031**	1.42	1.06 – 1.89	**0.019**
SM variety	1.38	1.10 – 1.73	**0.006**	0.79	0.58 – 1.08	0.142
weekly web duration	2.53	1.89 – 3.39	**<0.001**	0.43	0.29 – 0.64	**<0.001**
active weeks	2.41	1.69 – 3.44	**<0.001**	0.36	0.23 – 0.56	**<0.001**
Observations	1367			1367		
R^2 / R^2 adjusted	0.990 / 0.990			0.988 / 0.988		
AIC	1852.524			1865.074		
AICc	1854.527			1867.077		

S8.2 Table. Hurdle model with a negative binomial and zero-inflation negative binomial model for ChatGPT Adoption.

	Hurdle (NB)			ZINB		
Predictors	*Incidence Rate Ratios*	*CI*	*p*	*Incidence Rate Ratios*	*CI*	*p*
(Intercept)	1.11	0.62 – 1.99	0.713	1.41	0.91 – 2.19	0.122
gender	1.08	0.84 – 1.39	0.562	1.05	0.84 – 1.32	0.670
age	0.70	0.57 – 0.87	**0.001**	0.71	0.59 – 0.87	**0.001**
education	0.97	0.78 – 1.21	0.801	1.01	0.83 – 1.22	0.913
income	0.90	0.72 – 1.14	0.401	0.93	0.75 – 1.15	0.490
residence rural	1.10	0.88 – 1.38	0.386	1.10	0.90 – 1.34	0.368
income disclosure	0.79	0.65 – 0.97	**0.022**	0.81	0.68 – 0.96	**0.013**
need for cognition	1.22	0.91 – 1.63	0.175	1.22	0.96 – 1.54	0.100
writing skills	1.38	1.03 – 1.86	**0.034**	1.35	1.06 – 1.72	**0.015**
organisation skills	0.59	0.43 – 0.81	**0.001**	0.65	0.50 – 0.83	**0.001**
meeting skills	1.38	1.03 – 1.86	**0.032**	1.25	0.97 – 1.59	0.079
pol debate offline	0.85	0.66 – 1.09	0.201	0.91	0.74 – 1.13	0.385
pol online action	1.40	1.08 – 1.82	**0.012**	1.36	1.09 – 1.69	**0.006**
religious membership	0.78	0.62 – 0.98	**0.036**	0.86	0.71 – 1.05	0.137
SM variety	1.26	1.01 – 1.57	**0.040**	1.27	1.04 – 1.55	**0.017**
weekly web visits	1.53	1.03 – 2.28	**0.034**	1.49	1.05 – 2.11	**0.026**
active weeks	1.47	0.80 – 2.68	0.214	1.37	0.78 – 2.39	0.269
**Zero-Inflated Model**						
(Intercept)	0.05	0.04 – 0.07	**<0.001**	9.50	6.06 – 14.91	**<0.001**
gender	1.17	0.95 – 1.44	0.144	0.85	0.66 – 1.11	0.236
age	0.51	0.41 – 0.63	**<0.001**	1.82	1.41 – 2.35	**<0.001**
education	1.23	1.02 – 1.48	**0.028**	0.82	0.65 – 1.02	0.078
income	1.09	0.89 – 1.34	0.384	0.90	0.69 – 1.17	0.420
residence rural	1.19	1.00 – 1.43	0.054	0.84	0.67 – 1.06	0.152
income disclosure	0.79	0.67 – 0.94	**0.007**	1.21	0.99 – 1.47	0.067
children	0.81	0.66 – 0.99	**0.043**	1.27	1.00 – 1.62	0.051
employment	0.80	0.65 – 0.99	**0.042**	1.27	0.99 – 1.62	0.059
new media literacy	1.24	0.98 – 1.56	0.069	0.81	0.62 – 1.05	0.115
pol leaning	0.84	0.69 – 1.01	0.065	1.21	0.96 – 1.51	0.103
pol debate online	0.82	0.67 – 1.00	**0.047**	1.32	1.05 – 1.66	**0.020**
political knowledge	1.41	1.13 – 1.76	**0.002**	0.69	0.53 – 0.90	**0.006**
voted in election	1.32	1.01 – 1.72	**0.042**	0.75	0.56 – 1.00	**0.050**
SM Telegram	1.14	0.94 – 1.38	0.186	0.87	0.69 – 1.10	0.257
SM Facebook	0.80	0.65 – 0.98	**0.031**	1.34	1.04 – 1.73	**0.023**
SM variety	1.38	1.10 – 1.73	**0.006**	0.76	0.57 – 1.00	0.053
weekly web duration	2.53	1.89 – 3.39	**<0.001**	0.41	0.29 – 0.59	**<0.001**
active weeks	2.41	1.69 – 3.44	**<0.001**	0.43	0.27 – 0.69	**0.001**
Observations	1367			1367		
R^2 / R^2 adjusted	0.794 / 0.791			0.771 / 0.768		
AIC	1575.029			1583.833		
AICc	1577.145			1585.949		
