# Supplementary material for: Characteristics of ChatGPT users from Germany: Implications for the digital divide from web tracking data
Source: PLoS One. 2025 Jan 17;20(1):e0309047. doi: 10.1371/journal.pone.0309047 (PMC11741609; doi:10.1371/journal.pone.0309047)
Supplement: S2 File — (PDF) [file pone.0309047.s002.pdf]

Documentation of web tracking data collection based on Bilendi and Wakoopa service terms

This document is provided by Bilendi GmbH and should serve as a supplemental detail into data collection procedures for their web tracking services (also provided by Wakoopa).

1. Passive data specs

The following tables will be provided for each month:

- Web_pages_views = urls on desktop
- web_visits_id = visits on desktop (urls aggregated by visit)
- Mobile_views = urls / app on mobile / table
- mobile_visits = visits on mobile (urls / app aggregated by visit)
- App_metadata = app info
- Categories = domains info
- Youtube = YT video info
- Participant_meta_data = participant info. subsample for the period.

Each table will be provided as a zipped csv file.

- `web_pageviews.csv.gz`: all tracked urls on desktop-devices. One row corresponds to a hit on a specific webpage by a participant on desktop.
  - `p_id : participant identifier`
  - `url : url of the webpage`
  - `used_at : time stamp`
  - `active_seconds : time spent on the webpage in seconds (time-out of 3 mins if user inactive)`
  - `web_visits_id (equal to id in web_visits) : visit id to match with web_visits table`
  - `yt_video_id : if the url is a YT vieo, the youtube video id for match with youtube table, if not NA.`
- `web_visits.csv.gz`: all tracked urls on desktop-devices aggregated by visit on the same domain (urls on the same domain hit within 30 min span are put together). One row corresponds to a visit on a domain by a participant on desktop.
  - `id : visit to match with web_pageviews table`
  - `p_id : participant identifier`

- domain : domain of the webpage
  - used_at : time stamp
  - duration : time spent on the webpage (sum of active_seconds on corresponding urls) in seconds
  - pageviews : number of pages visited

- mobile_views.csv.gz: all tracked views on mobile devices. One row corresponds to a content (webpage or app) hit by a participant on a mobile or tablet.

  - mobile_visits_id (equal to id in mobile_visits) : visit id to match with mobile_visits table
  - p_id : participant identifier
  - url : url of the webpage (or NA if app)
  - domain : domain part of the url (or NA if app)
  - app_id : app identifier for matching with app_metadata table (or NA if url)
  - used_at : time spent in seconds
  - connection : wifi or cellular
  - duration : time spent on the webpage / app (in seconds)
  - yt_video_id : if the url is a YT vieo, the youtube video id for match with youtube table, if not NA.

- mobile_visits.csv.gz: all tracked urls on mobile / tablet -devices aggregated by visit on the same domain (urls on the same domain hit within 30 min span are put together, plus app use. One row corresponds to a visit on a domain by a participant on mobile/tablet or the use of an app
  - id : visit to match with mobile_visits_id in mobile_pageviews table
  - p_id : participant identifier
  - domain : domain of the webpage
  - used_at : time stamp
  - duration : time spent on the webpage (sum of active_seconds on corresponding urls) in seconds
  - pageviews : number of pages visited

- app_metadata.csv.gz: metadata on mobile apps. One row corresponds to an app and the associated information.
  - app_id : app identifier for matching with mobile_views
  - app_n : name of the app
  - app_os : iOS or Android
  - app_cat : app category as per store

- categories.csv.gz: webshrinker.com categories for domains. One row corresponds to a domain and the associated information.
  - domain : domain for the website
  - category : list of relevant webshrinker categories (as per webshrinker.com)

- youtube.csv.gz: youtube videos infos. One row corresponds to a video and the information about the video retrieved thru the YT API.
  - video_id : youtube video for match with

- title : video title
- tags : video tags
- channeltitle : YT channel on which the video is published
- viewcount : number of views
- description : description of the video

- panelist_metadata.csv.gz: metadata on participants that has been active in this period. One row corresponds to a participants and its socio-demographics.
  - p_id : participant identifier
  - birth_day
  - birth_month
  - birth_year
  - gender
  - Federal State
  - Family Status
  - Household – Number of children
  - Education – School education DE
  - Employment – Career Status
  - Size of place of residence
  - Household income – Euro
  - age
  - property – Own estate
  -

2. Measurement procedures

a) Desktop metrics

The desktop tracker is a stand alone application that captures the full URLs the participant visits. Only the behavior in the supported browsers is measured.

What do we measure on desktop?

- Participant id
- URL visited (both domain and path)
- Time stamp
- Duration

Measurement specifications

Once installed, the tracker application is running in the background of the device. It checks every two seconds for activity on the desktop. When the participant is using the browser, it scans the active browser and tracks the URL of the page the participant is using. When the participant uses more than one browser or more than one page in one browser, only the URL in the active tab is tracked.

The scan is repeated every two seconds. Every time an URL is tracked, the tracker application checks whether it is equal to the previous URL. If the URL is different from the previous URL, the tracker registers this as a new pageview and stores it together with the time stamp.

If at the moment of the scan the URL is not complete - for example when the participant did not finish typing the URL - the tracker application also tracks these URLs. Yet, before storing any pageviews it is checked whether the URL is valid (e.g., contains a ".").

When the user switches tabs, the active tab changes. The tracker application tracks the URL in the active tab and compares it to the previous URL. Because it is different form the previous URL, it registers a new pageview. Using this method each new URL is registered as a new pageview. However there are two exceptions to that. The first exception is when a participant visits three URLs in the interval of two seconds, the middle URL is not tracked. So when someone is browsing really fast, the URLs that are seen less than one second are not tracked.

The second exception is when a page is refreshed (manually or automatically), the page is reloaded, but the URL of the refreshed page does not differ from the previous URL. Therefore, the refreshed page is not stored as a new pageview.

Visits

At the end of each day at 0:00, the data is exported to S3 to be processed. The source of the data are pageviews, but after each day the data is reformed and the pageviews are grouped into visits. A visit is a series of pageviews within a group of URLs visited by the same participant. Between each URL there is a period of no more than 30 minutes. Calculating the visits will result in a list of visits with each visit having a: participant, group of URLs, time of visit, total number of seconds and the total number of pageviews. Part of the definition is group of URLs. The groups are defined by a domain (e.g., facebook.com or youtube.com) or address (e.g., facebook.com/events or youtube.com/history).

Domain visits

Domain visits are calculated by default and they apply the definition to a specific domain. A domain is the main part of the URL. For example, the domain of `www.youtube.com/watch=12334` is `youtube.com` and the domain of `www.facebook.com/events/list` is `facebook.com`.

A **domain visit** on youtube.com would be:

URL	Time
http://www.youtube.com/watch?v=IGFh70WxNKA	13:44
http://www.youtube.com/results?search_query=rolling+...	13:55
http://www.youtube.com/watch?v=jgWUi-ozMAU	13:56
http://www.youtube.com/results?search_query=amelie...	14:01

Note: For technical reasons a visit cannot span multiple days. All visits are cut off at 0:00 pm (in the time zone of the participant) and a new visit is created for the new day.

Unique visitors

Tracker based measurement is user centric. The participants install the tracker on their devices. This way all data can be lead back to single persons. Also when participants are using multiple devices (e.g., desktop, smartphone and/or tablet) the data that is gathered can be connected because each participants has it's own unique id.

Unique visitors are calculated from the visits data. Each visit contains the user id, the number of pageviews, the start and end time of each visit. To calculate the number of unique visitors on a certain domain in a certain time period, the number of unique id's in the selected visits is counted.

b) Mobile Views

Introduction

The mobile data is offered as Mobile Views. The Mobile Views are created via an advanced algorithm that uses cleaning, filtering and processing. On all versions of Android we are able to report full length URLs for both HTTP and HTTPS. On iOS we are also able to report full length URLs for HTTP, while subdomains and top domains are reported for HTTPS.

Our algorithm works for all Android versions and on iOS. However, in iOS there are differences in the steps that have to be taken in order to end up with clean data.

For Android we measure both app and web usage straight from the OS (using the Accessibility API for Web measurement from Android 6.0 onwards). No additional cleaning is required.

On iOS we use a proxy to measure App as well as Web behavior.

For proxy data *all* requests done by the device are being captured. So when a page is loaded, we not only see the request to actually load the page, but we also see requests for loading all assets on the page like images, scripts, content delivery networks etc.

On iOS we not only see all requests from a website, we actually capture *all* requests the device does. For example background processes of the device, syncing with iCloud, retrieving security settings, retrieving e-mail etc.

This article explains how we create our end product, the Mobile Views, from this raw proxy data in iOS.

In order to clean the proxy data, we start off by classifying and tagging all data events. These tags can then be used in the processing.

How do we move from raw data to classified and tagged data in iOS?

The first step in the data processing is to identify the data that is valuable for reporting and what data should be filtered.

The processing starts off with an initial step to make the data readable:

- identify the client-id, the user-id and the device-id (based on device authentication)
- parse the captured URLs into subdomain, domain and path
- convert the captured server time to the local time of the device

Classification of the data events

After the initial processing we classify all events. This allows us to filter the events that are not of importance for the reporting, such as system applications, background applications but also assets loaded on a website or sites that are on our blacklist of domains and patterns (CDNs, ad networks, etc).

Processing our iOS proxy data into the actual mobile pageviews

After the step of classification of the events the actual processing starts. This processing will turn the raw proxy data into data at the Mobile Views level.

All data is collected with the following principles in mind:

- The device must be active
- The process reported on must be in the foreground
- The duration starts counting when the process is active (in the foreground)
- The duration stops counting when the process stops, goes idle or moves to the background

These principles are applied for all devices, regardless of operating system. On Android and Desktop we are able to measure the above directly, while on iOS we need to process and calculate this from the captured data.

The algorithm we use for this on iOS is as follows:

- Define when a device is active, by creating activity blocks from the proxy data
- Create groups from the activity blocks
- Create views from the groups

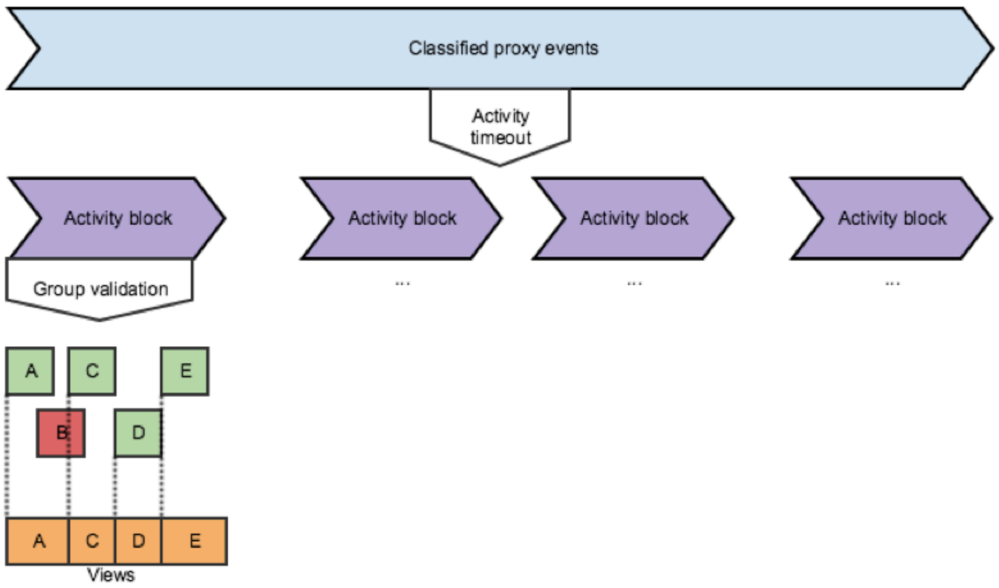

Create activity blocks of the proxy data

To define whether a device is active (one of the definitions of a view), we transform our proxy data into activity blocks. This is done by using a timeout on the data coming in. If we do not see data coming in for **125 seconds**, we define the device as inactive.

The timeout was defined after elaborate research on different timeouts and the impact on the numbers of views and amount of duration this has had. If you are interested in the research, you can always contact us for more information.

Why are we using a timeout?

The proxy that is configured on the device will measure **any** internet activity on the device. These activity events do not indicate exactly when the device was active or inactive. It can happen that the device makes a connection to the internet without the device being actually active. For example, e-mail synchronisation in the background. On the other hand, it can happen that an application loads its information all at once, but the app keeps active for a longer time without creating any data streams.

With the timeout we ensure that when this happens we report on the correct time the device was active.

Creating groups of valid data out of the Activity blocks

After we have defined when the device was active, we clean the data from unwanted events. The classification from the first phase is used here.

The groups are defined by looking at the apps that have created the events. All background or system apps are filtered from the data and the remaining App and Web data (data produced by browsers) are treated seperately.

The Web data will get another layer of filtering. First we remove blacklisted URLs and assets (also based on the tagging from the first phase), and after that the URLs are split into validated groups based on:

- https domain (https domains need to show more requests in order to be seen as valid)
- referer (see this wikipedia page for more information)
- url if valid

Create views out of groups

When the groups are defined and validated we create pageviews out of the groups. We first create 'sub-groups' out of the groups, based on a timeout of 30 seconds. This means that if somebody visits a URL and does not show activity on this URL for 30 seconds, and then goes back, this is seen as a new subgroup.

These sub-groups are then transformed into views with an attached duration based on:

- The start timestamp (the first entry in the sub-group)
- The end timestamp (the start timestamp of next sub-group)
- Last sub-group (the last entry in the sub-group)
- The duration is the difference between the start and end timestamps

Result

The whole process described in this article will run overnight and will produce an easy to use data file that is available to you the next morning.
